# The GP’s a stranger: an interpretive phenomenological analysis exploring patient experiences of changed access to primary care in the management of long-term conditions

**DOI:** 10.3389/frhs.2025.1473680

**Published:** 2025-04-29

**Authors:** Sandra Walker, Tansy Daniel, Mediha Yildizcan, Jennifer Karen Roddis

**Affiliations:** ^1^ School of Health and Care Professions, University of Portsmouth, Portsmouth, United Kingdom; ^2^ School of Pharmacy and Biomedical Sciences, University of Portsmouth, Portsmouth, United Kingdom

**Keywords:** interpretive phenomenological analysis, patient-centred healthcare, long-term condition, qualitative, trust, expert by experience

## Abstract

**Introduction:**

Self-management is promoted as a mechanism for those with long-term health conditions to manage their condition day-to-day. Changes in access to primary care in the UK have led to an increased patient burden and reduced access to care.

**Methods:**

This exploratory study considered the impact of such changes for those managing long term physical and mental health conditions. An interpretative phenomenological analysis approach was adopted. Interviews were conducted with eight individuals affected by long-term physical and/or mental health conditions.

**Results:**

One overarching superordinate theme was identified as significant to all participants: The GP's a stranger. This superordinate theme was fundamental to five lower order themes: Role of GP; Fighting to gain access; Dismissed, depersonalised and devalued; Resourcefulness borne of desperation, and “There was something wrong”, which offered insights into the experiences of participants.

**Discussion:**

Those living with long-term conditions often know when they need to seek additional healthcare support however they shared multiple barriers to accessing this support when needed and reported that the lack of relationship with any health care professional in primary care affected their ability to trust any care advice they were given. Considerations of a new way of operating within a changed paradigm of primary care are explored.

## Introduction

Prior to the Covid-19 pandemic, difficulty in accessing primary care and changes in practice, with receptionists taking a more prominent role particularly in protocol driven triage, were identified as some of the factors leading to increased use of Emergency Departments (1, 2) These changes occurred in a context of continued austerity in the public sector, a reduction in the number of GPs plus an ageing population with increasingly complex comorbidities (3). An increase in patient burden (4) is noted as a result of these factors and those with ongoing physical and mental health long-term conditions (LTCs) are likely to be particularly adversely affected as the majority of LTC care is carried out in primary care (5). As the GP is known to be an important part of the network of a person managing a long term condition (6, 7), changes in accessibility and practice are likely to have a major impact on this group. Payne et al. (3) highlight the unintended dehumanised and fragmented care that has resulted from increased digitalisation, extension of roles and protocolisation of primary care practices.

In England alone there are more than 15 million people who are managing a long term health condition with this number expected to increase (8). In the UK, 90% of people experiencing mental distress are managed in primary care (9), with only a small percentage of people qualifying for specialist mental healthcare. The DoH points out that “Mental ill health is the single largest cause of disability in the UK” (10). Services in the UK are primarily available via primary care (IAPT services and medication) for those categorised as experiencing “minor to moderate” mental ill health (9). These are usually people suffering symptoms linked to depression and anxiety, which can be very disabling and seriously affect work and family relationships [ibid (11)].

Self-management is promoted as a mechanism for ensuring those with long-term conditions are able to manage their condition day-to-day (12) with access to primary care being a key feature required for the success of the approach (13). Additionally, a key element of self-management is the patient knowing when they are no longer able to manage a condition alone, and that additional care, advice or support is required (14) and the expectation is that they will be able to access this from their GP. Taylor et al. (15), in a synthesis of interventions supporting self-management for people with LTC, demonstrated the importance of understanding patients’ knowledge and beliefs about their LTC and noted that supporting self-management is inseparable from high quality care in LTCs.

Global evidence indicates that, since the pandemic began, patients have struggled to gain in-person access to GPs or other practice staff (16). Whilst there has been a move towards online or telephone consultations instead (17, 18), unmet needs can result, including being unable to obtain prescription medication and essential medical equipment, together with delays in investigations and diagnosis and consequent increased severity of conditions (19, 20). In a project which sought to understand whether access to healthcare for those experiencing health inequalities, including those with LTCs, had been exacerbated further due to the pandemic, Topriceanu et al. (21) found that those with chronic illnesses were more likely to have cancelled medical appointments and to require a greater number of care hours.

The literature demonstrates some of the unmet needs experienced by patients accessing healthcare online or by telephone, and highlights the potential for changes to primary care practices to result in patients experiencing dehumanised and fragmented care. However, there is a lack of evidence around the impact of these changes to access to primary care on patients’ wellbeing and self-management of their long-term condition, across the entire experience of accessing care. This small study begins to consider this.

### Aim

The aim of this project was to explore people's experience of the recent changes in engaging with the GP and how these may have impacted the wellbeing of people with, and self-management of, LTCs.

## Methods

### Methodological approach

Semi-structured interviews were undertaken either face-to-face or online via Zoom. The study was underpinned by an interpretative phenomenological analysis (IPA) approach, which informed interview questions as well as the analysis. Interpretative Phenomenological Analysis (IPA) was chosen as the best means of exploring the patient experience in the particular circumstance of accessing primary care. Although originating in psychology, this approach is now increasingly used in health and social sciences (22). A founding principle of phenomenological inquiry is that an experience should be looked at from the perspective of the way it occurs and on its own terms.

IPA is a qualitative approach which aims to provide in depth examinations of lived experience (22). It produces an account of personal lived experience in its own terms rather than one prescribed by pre-existing theoretical preconceptions and it recognises that this is an interpretative endeavour as humans are sense-making organisms. IPA is a particularly useful methodology for looking at topics which are complex, ambiguous and emotionally laden (23).

### Recruitment

Posters were circulated via local organisation mailing lists asking people with a professionally diagnosed LTC such as asthma, diabetes, hypertension, bipolar disorder to make contact with the researchers either by phone or email. These included support groups for those with LTCs, student societies and groups. The poster stated the aim of the study (“to look at whether the recent changes in access to GP services is impacting on the wellbeing of people with, and self-management of, long term conditions”), it indicated that people with both physical and mental health LTCs were welcome to come forward and outlined details of the two main researchers and the two student researchers who would be involved in the study. Participants self-nominated to take part by email or phone and were contacted by SW or JR in order to discuss participation.

Participants were people with professionally diagnosed LTCs who are self-managing in the community and would ordinarily be using primary care services with occasional input from specialists in secondary care services. Specific inclusion and exclusion criteria are shown in Table 1.

**Table 1:** Inclusion and exclusion criteria.

Inclusion	Exclusion
Adults 18 +	Under 18s
English speakers	Non-English speakers
People with diagnosed LTC	People with self-diagnosed LTC
Mental capacity intact	Mental capacity challenged

### Data collection

Those responding to the call were contacted by telephone to share more information about the project and to ascertain suitability for the project. They were provided with participant information and the opportunity to have any questions answered. Once participants agreed to take part and were confirmed as meeting the inclusion criteria, a mutually agreeable date for interview was set to take place either at the university or over zoom depending on participant preference. The interviews were recorded and transcribed verbatim. The interview recording, transcript and the interviewers’ field notes constituted the data which was analysed using the framework of IPA.

### Data analysis

Data analysis began as soon as the first interview was completed. The interviews with the participants were all analysed and themes identified. The following process was undertaken by both researchers and a student researcher for each participant. The interview was listened to along with the transcript and any corrections made, then the text of the transcript was read and re-read whilst listening to the recording to ensure immersion in the data. One by one each transcript was examined to get a clear picture of the experience of the individual. The transcript was already numbered line by line and phrases directly related to emergent themes were extracted and further considered. At this stage the themes related to the individual cases, however, as Smith et al. (22) point out it is likely that emergent themes that occur across cases will be starting to emerge here but the process of looking for patterns across cases is resisted at this stage to ensure that each case is considered as fully as possible before turning to look at patterns and inter-case connections.

Once the initial analysis of each case was complete, the themes that had emerged were considered in a cross-case analysis which was completed in meetings of the whole team. One key emergent theme became clear for the whole cohort although when illustrating these themes, the text used still comes from individual transcripts. Pseudonyms are used to protect the identity of each of the participants and no names used in the interviews of people or places were mentioned throughout or in the transcripts from which the quotes are taken. Analysis of the interviews revealed one super-ordinate theme (SOT), with a total of five lower order themes (LOT). A SOT is one which usually applies to each participant within the group although it may look slightly different for each (22). A LOT is one that informs a major theme and there may be several of these in each major theme (ibid). In order to reinforce rigour and ensure remaining close to the experience of participants, reflexive notes were kept by each member of the research team which included consideration of the insider status of each as a user of primary care, two of the team having current LTCs.

### Ethical considerations

Ethical approval was sought via the University of Portsmouth Faculty of Science and Health Ethics Committee (SHFEC 2022-047) and acquired in June 2022.

## Results

The participant cohort included people with both physical and mental health LTCs. Most indicated that they had more than one LTC. Participants were adults (over 18) self-referring from community sources, therefore the sample was purposive in nature. The aim was to include up to 10 participants due to this being an unfunded, exploratory project, together with the intense nature of the IPA process. Fourteen individuals made contact to find out more about the project, and eight took part. Of the six who did not progress to interview, the communication from enquirers simply stopped in 5 cases and in one case a potential participant withdrew due to information technology issues. All participants that came forward met the inclusion criteria. Participants (6F, 2M, Table 2) all had LTCs that were being managed via primary care with one exception whose mental health issues were primarily managed in secondary care with the GP acting as a medication prescriber alone. Conditions reported included: Type 2 diabetes; Klippel Trenauney Syndrome; shattered tibial plateau; awaiting knee replacement surgery; hypermobility spectrum disorder; fibroids; lipodema; lymphoedema; plantar fascitis; deafness; labyrinthitis; vertigo; depression; anxiety; bipolar disorder; autism; anxiety depressive disorder. Participants had a mean age of 55.75 and all were White British. The geographical location of the participants was not gathered, beyond being UK based, as this was a small study and there was no capacity to explore local service provision as part of the work.

**Table 2:** Participant demographics.

Participant	Sex	Age	Work situation	Education level	Relationship status	No. of long-term conditions
P1	M	60	Employed FT	College	Single	Five
P2	F	58	Employed PT	University	Married	One
P3	F	75	Retired	University	Div/Sep	Three
P4	F	20	Employed PT	University	Single	Two
P5	F	61	Unable to work due to disability/ill health	University	Single	Six
P6	F	59	Unable to work due to disability/ill health	College	Div/Sep	Three
P7	F	50	Unable to work due to disability/ill health	Secondary School	Single	Three
P8	M	63	Unable to work due to disability/ill health	University	Married	One

**Figure 1:**
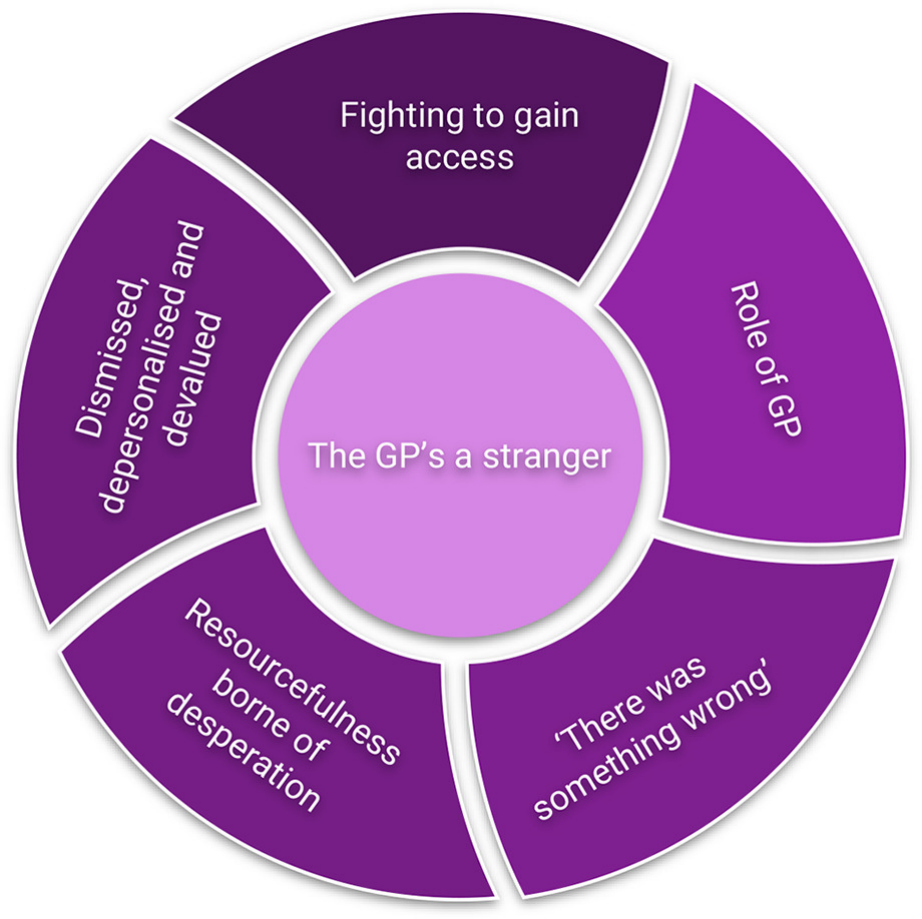
Superordinate and lower order themes.

Following analysis, one SOT emerged that was common to every participant: “The GP's a stranger”, and five LOTs were identified for which the SOT was agreed to be fundamental; Role of GP; Fighting to gain access; Dismissed, depersonalised and devalued; Resourcefulness borne of desperation, and “There was something wrong”. Please see Figure 1 for the SOT and LOTs identified.

### “The GP’s a stranger”

This SOT was broken down into five LOTs as outlined above. This superordinate theme was considered to be of such fundamental importance that it influenced and impacted all of the LOTs significantly. The LOTs are outlined in order below. All participants described how limited their contact was with their GP, and how they perceived their (sometimes non-existent) relationship with their GP. This relationship affected the willingness of participants to seek medical help. In some instances, they compared their current experiences with previous positive engagements with their GP and practice.

“I always felt that it would be possible to be seen quickly if necessary … I do feel to quite a degree that there's been a loss in interpersonal connection really, that I think I'm probably less likely to go than I was because I felt that I would be able to be seen quickly, and it would be a good interaction. Whereas now it's very much potluck…it's a non-human relationship to a degree … feels like you're dealing with some sort of conglomerate … It doesn't quite feel like the healthy relationship it should be … it's feeling disjointed and dysfunctional … the GP's a stranger, I wouldn't know them” (P1)

“Some of them [GPs] have no idea… they just don't know me, and it's yeah, a lot of work. I mean that they seem nice enough. It's just like, makes appointments about ten times longer or more difficult … It puts me off seeing my GP to a degree” (P4)

“I haven't got an actual GP. I did have an actual GP but actually unfortunately, she left at the time. So I've just had different GPs really”. (P7)

This reported loss of relationship resulted in a lack of trust in their healthcare professional (HCP), not just the GP, as many reported seeing other HCPs for things they might have previously seen a GP for, for example, diabetes. Additionally, as a result of the limited involvement of GPs in their LTC management, participants described having a limited—or non-existent—relationship with their GPs. This made it difficult to seek support when they needed it.

### LOT 1: role of GP

This LOT refers to the role participants saw their GP having in the management of their LTC, of the eight participants, only three had met their named GP and only one participant (P6) considered their GP to have a positive role in the management of their LTC. Of the remaining seven, two stated that the role involved prescribing medication only and the remainder felt the GP had no role in helping them manage their LTC.

“So my GP doesn't really have any role in medication except to write the prescriptions” (P8)

“Absolutely none, I've never met her. I just know her as a name. The GP had no function at all.” (P3)

Participants described a lack of continuity in their dealings with their GP which often left individuals unsure about who they should listen to, as they did not always get the same answer for the same issue:

“But what I find is that one GP will tell me one thing, and then another GP will tell me something else, so sometimes I get a bit confused with it” (P7)

Individuals also spoke about seeing different GPs for each appointment, resulting in them having to repeat their medical history each time, and receiving inconsistent care as a result:

“But you never get the same GP so you never get that continuity, or then you have to explain everything, every time you go.” (P2)

The lack of consistency led to participants feeling ignored and overlooked, and they worried for the safety of other patients:

“I understand that GPs are our gateway service… It's not supporting the patient. With physical long term conditions, it needs to go to the right area to get managed with somebody overlooking them … People with long standing conditions are being ignored. And I mean, all long standing conditions are being ignored. Who's monitoring elderly, frail people from within that surgery?” (P5)

Some participants described incidents where the GP did not know about the LTC they had and, from the participants perspective, rather than trusting their patient knowledge, the GP searched the internet for answers.

“Oh, I've never heard of that. Let me google, you know, so it's that kind of thing” (P2)

There was evidence of entrenched cultural expectations, whereby the patient automatically trusted healthcare professionals:

“We've got confidence in them which counts for a lot.” (P8)

However in the same interview, this participant talks about behaviours from a GP surgery designed to limit patient access which suggests service factors at work that influence the capacity to create a relationship between GP and patient:

“One manager said that they redid their appointment system every six months to keep the patients guessing.” (P8)

Overall, GPs played a limited role in the management of participants’ LTCs, which meant that it was not possible for a relationship to be built, founded on trust, with their patients.

### LOT 2: fighting to gain access

Participants found it difficult to gain access to healthcare through their GP practice—whether this was access to a GP or to another relevant professional. This began at the point of seeking an appointment and continued during appointments. Several participants spoke of how challenging it was simply to book an appointment, with various routes to do this, not all of which were open to everyone.

“well, it seems to be becoming, jump through, you know, two hoops, three hoops, walk five, six hoops, it just seems more and more tricky to even access … I mean, just talking to the human being even if that's the receptionist … a lot of it, you know, is you can go to this link. And well, I'm not online at home, my phone … I can get online if I go to the library, but the library is further away than popping into the practice.” (P1)

“I have had the problem where it's quite hard to get through to a GP and then I've had it before where I've actually stayed on the link until I've been number one in the queue and then they actually haven't picked up the phone.” (P7)

Many participants described how the staff they met either on the phone or in person in surgeries acted in ways which created a barrier to accessing help.

“You go to the practice and you walk up to reception and say, Can we get an appointment. No, you have to ring this number. I'm here. What is the sense?” (P1)

In referring to the person who was first point of contact (sometimes referred to by participants as the receptionist), P5 demonstrates a tension between the name used at their practice—“caregiver”—with their experience of speaking to them:

“A caregiver, who are not, as they always tell you, when you ask them a question, are not medically qualified, they're just a call centre … because that's who you talk to no matter whatever number you go through you talk to a caregiver who don't do care.” (P5)

Participants experienced the processes of the system as challenging and they did not feel that they made sense. This contributed to the feeling of not being understood by their general practice and thus that the system was failing to support them in seeking healthcare for their LTC.

### LOT 3: dismissed, depersonalised and devalued

Once individuals had managed to get an appointment, they often did not see a GP but another HCP. Whilst they often understood the rationale for this, participants reported that they felt dismissed, depersonalised and devalued as a result. Unfortunately, whilst it may be appropriate for patients to see someone other than a GP, this had not always been communicated to patients:

“there's been a move in primary care for ages to move the work down to the least qualified person … then the GP can concentrate on the expertise that they've spent a lifetime acquiring” (P8)

“It may not always be a GP that you need to see, you may be referred to somebody else. and that's fine, you know, as long as they have the medical experience. I don't mind seeing other people, you know, the other nurses as well.” (P6)

“come in for your diabetic review and it's been the nurse doing that … I think she's taken over from the GP in that sense. So I'm not sure that I think that's necessarily a good move … I guess you feel downgraded. And, you know, you can understand things have to be prioritised and that's really valid. But I guess it does have an effect on how you feel you're seen by the practice … don't want to be, feel a lesser priority, I guess” (P1)

Sometimes people also felt that they were not being listened to, and that this was exacerbated by the move to telephone appointments:

“I've had it where I've actually wanted to say more about how I'm feeling, about my health condition. And before I've got time, they've put the phone down and I'm thinking well, you know, I haven't had enough time to say what I needed to say … ” (P7)

Telephone appointments were also challenging for those who struggled to speak about their health conditions and relied on their facial expressions and body language:

“I feel like it's made it more difficult, um, I, I'm not the best at talking, uh, so I communicate quite a lot more with expressions and, um, body language, so it's easier to have a face to face appointment, and otherwise it's made it quite tricky to try and explain things to people” (P4)

Similarly, those whose own health conditions meant it was challenging to speak on the telephone described how they felt depersonalised:

“I did get to speak to a receptionist. But I have an added problem … in that I'm quite hard of hearing. It's very difficult. And I’ll keep saying, and I've had some very rude comments, I would say, from some of the receptionists, I said, Can you please speak up? Well, I’m speaking as loudly as I can!” (P3)

“you get defined on the phone, especially if you have mental health, you get defined as hysterical. And I know the minute they hear a woman's voice, hysterical” (P5)

This LOT further emphasised participants’ lack of trust that the healthcare system and those working within it were acting in the best interests of the person with a LTC.

### LOT 4: resourcefulness borne of desperation

Some individuals spoke about finding ways around the system that enabled them to manage their long term condition in ways they felt worked for them. Such mechanisms may go against expected protocol from the HCP perspective, and appeared to be a result of difficulties in access (see above):

“bypass the system a little bit. So I um email them to contact my nice GP who normally gets back to me. So unless it's really urgent, I just like, wait until she does that.” (P4)

Whilst this participant described a “nice GP”, this was in light of this being the only individual at her practice that she felt would get back to her, and she acknowledged that this GP was not always the appropriate person to contact by recognising that this involved bypassing the system.

In some instances, this was in order to follow the advice of other HCPs:

“I said, I've just spoken to 111. I need to see a doctor today. I said, heart palpitations. And at that moment they had really calmed down and she asked me, have you got them at the moment? And I went, No. She said, Well, it's not an emergency then. I said, Well, 111 has told me. She said, Well, it's not an emergency … I got so angry and said right, they've happened just now. And she said, What have they just come on? And she knew I was lying. And I knew I was lying. But that's the only way I got to see a doctor that day” (P5)

Others used their professional knowledge to inform the way they managed their long-term condition:

“getting a blood test might not be on the right, might not be on the right day for my Warfarin but because they'd tend to be booked up for a month in advance … that's fine. Because I'm a nurse, … I could control my medication” (P2)

Participants found ways to work around the system when it was not working effectively for themin order to support their self-management.

### LOT 5: “there was something wrong”

This LOT is characterised by both risk events and fears resulting from patients filling in the gaps where there is missing information. We have referred in previous sections to the emotional and psychological outcomes of changes to access. Participants also spoke of the health outcomes that had resulted from being unable to access their GP in connection with their long term condition, in some instances these had been life-changing or even life-threatening.

There were some significant risk issues which participants believed occurred as a direct result of difficulties accessing the service:

“I wouldn't have had a stroke if I'd had proper access to my GP” (P2)

“So there's been some funny little things about the scheduling of the tablets, medication…. then it was a matter of me talking to the pharmacy, talking to the receptionist or whoever practice and the practice getting back to the pharmacy, and then should I contact the….? So it was a bit of a triangle?..for a time it was slipping quite badly.” (P1)

“then my GP at the time said, try cutting it in half. You know? Yeah. and then somebody said, you mustn't do that. I don't know who was it? Oh, it must have been this older person's psychiatrist who said, No, you can't cut them in half” (P3)

Lack of communication with test results combined with the lack of relationship with the GP was reported as creating fear of unspoken or missed risk issues for some participants. This gap sometimes led them to create information to fill the gap themselves in an attempt to explain things. The lack of communication in this area led to confusion and distress. In the following quotes the participants were not made aware of results of the tests they had had and described making up stories about it which could be very anxiety provoking in themselves.

“It's a complete mystery. And so because it's a mystery, it's the unknown and fear of the unknown is one of the major fears. And that doesn't help with mental health either.” (P1)

“it is quite a stressful time just going for scans and thinking one way, they're not finding anything but on the other hand you think well, what's wrong when you keep having the scans and of course what's causing the [symptom]” (P7)

Adverse incidents and fears resulting from being unable to receive care in relation to their LTC led further to a lack of trust between participants and the healthcare system they rely on to manage their LTC effectively.

## Discussion

Changes to the primary care system in the UK were clearly required, resulting for example in super partnerships and integration of practices into Primary Care Networks, due to increasing demand and thus pressure on services, decreasing patient satisfaction and increasing health inequalities (24–26). This study has identified a number of negative impacts resulting from these changes for those with LTCs. These include: patients feeling that they have no relationship with their GP; a subsequent lack of continuity in advice provided to patients; a lack of trust between clinicians and patients; patients experiencing issues with accessing their primary care provider; the role of the reception team in preventing access to healthcare; patients feeling depersonalised and devalued; desperation on the part of patients leading to resourcefulness; patient perceived risk outcomes on physical and mental health as a result of these impacts, as well as fear and misunderstanding.

The literature demonstrates the relevance of a good patient-clinician relationship and the importance in particular of trust within the dyad (27). Although none of the participants mentioned the word “trust” directly, they all described issues such as not knowing the GP, lack of continuity, not feeling they were listened to and so forth, all of which contributed to the overarching sense of erosion of trust in their HCP. These are known to be issues which contribute to the relationship between HCP and patient, and key to maintaining health (28). Hewitt-Taylor and Bond (29) found that patients with diabetes considered it important to have a good relationship with doctors. More recently, Budge, Taylor and Curtis (30) identified that patients who felt their healthcare professionals did not know them experienced frustration. Similar to our findings, their participants valued continuity of care which allowed them to address the current issue rather than repeat the history of their condition over and over again. Both patients and GPs valued continuity of care which enabled ongoing relationships (31). A recent review of trust in healthcare (32) found that the majority of papers in this area relate to patient trust in healthcare professionals (*n* = 499) with a small number of articles focusing on trust exhibited by HCPs towards their patients (*n* = 11). Grob, Darien and Meyers (33) suggest that this could be the result of a paternalistic approach to medical care—patients should simply accept that the doctor knows best. The participants of this study demonstrated difficulty in accepting this from someone they found to be a stranger. There are implications for clinicians trusting their patients: such trust is mutual and, where doctors trust their patients are giving accurate reports of their health concerns, this can engender trust from the patient (33).

People who manage their LTC day to day often know how to deal with their condition and when to ask for help if they feel something is wrong (7) and the expectations of this group are minimal day to day. Therefore, in a person-centred system, the general practice team need to trust the patients and minimise barriers in the way of accessing the support needed to prevent deterioration. This study demonstrates how existing barriers, such as accessibility, prevent patients from accessing necessary support from practitioners, thus restricting opportunities to build a trusting relationship with primary care staff and particularly their GP. The relationship between the patient and the HCP has long been known to be important in influencing patient satisfaction and adherence to treatment regimes and is a clear quality indicator for services (27, 34). The findings of this study indicate that the patient experience of their relationship with primary care has deteriorated and this warrants further examination. Participants felt that they and their health concerns were dismissed, leading to a feeling of being devalued. This exacerbated their lack of trust in the services they rely on to manage their LTC.

The landscape of healthcare has changed considerably over the last decade. Shutzberg (35) suggests a new paradigm for the doctor patient relationship is emerging which resonates with the findings of this study. They describe the three main archetypes of the role of the doctor as parent (paternalistic services), partner (collaborative services) and service provider (consumerist) which have historically been experienced in healthcare. They outline a new paradigm of “bureaucratic parsimony” which has rendered both the doctor and the patient powerless in the relationship and suggests the opportunity for a new way of thinking about the doctor patient relationship. Trust is a poly-valent, psychosocial phenomenon (36), operating at different levels. The issue of trust on a personal level will inevitably be intertwined with the expressed needs of the patient and the often tacit needs of the professional. Thus the mechanisms of the organisation together with the individual circumstances involved in creating trust are separate from a desire to be trustworthy and demonstrate trustworthiness convincingly (36). Thus trust in the individual practitioner is always influenced by the expectations of the operation of the system within which the individual sits, in this case the Primary care system. The findings of this study speak to the theorising of Shutzberg (35), who suggests a new opportunity for doctors and patients to create a solidarity as comrades in order to work together to meet both the bureaucratic and austerity demands which have changed the face of healthcare.

This group of participants appeared to lack trust in their health providers and reported the belief that part of that issue resulted from the professionals not knowing them and trusting their judgement. Within the system there is an expectation of mutual trust without an enabling environment required to allow this trust to develop. In an increasingly complex and time-poor primary care system, HCPs and patients working in a mutually trusting partnership offers an opportunity to manage LTCs in a safer and truly person-centred way. The conditions needed to allow this to occur, in the current healthcare climate, require further consideration and research in order to create recommendations for practice. The changing models of primary care and the introduction of new roles means that where patients may previously have had the expectation that they would see their GP, it is now much more common that another HCP attached to the practice may be more appropriate. Therefore, it may be necessary for practices to promote this possibility and make patients more aware so that their expectations can be revised with commensurate lessening to impact on their LTC management.

### Strengths and limitations

This was an exploratory study which of necessity meant that the number of participants was small. Despite this, participants described experiencing a range of health conditions, including both physical and mental, and most experienced more than one condition. Participants were recruited from across the UK. Our study was not large enough to identify links or discrepancies between participants’ location and their experiences, though these did not appear to differ. There was a lack of cultural diversity among participants and only two male participants came forward to take part which limits findings. It is possible that the nature of the study encouraged those with negative experiences to come forward. However, some participants spoke of previous positive experiences with their GP and expressed support for their local GP practice. Others had themselves worked in healthcare and understood the challenges faced by the sector.

Given the increasingly complex nature of general practice, where it is possible that even people working from different locations within a group do not know each other, recommendations for research include identification of ways in which patients and clinicians can be supported to rapidly build trust in one another, in particular the adoption of an underpinning assumption that the patient with a long-term condition is likely to know their condition and themselves. Work to explore the effects of modern primary care practice is required to investigate the effect on the doctor patient relationship of bureaucratic parsimony and the opportunities it presents for a new relationship to develop.

## Conclusion

We undertook an exploratory study to understand the impact of changes to primary care on the experiences of people with long-term physical and mental health conditions. Our findings demonstrate a predominance towards negative impacts, including that the GP can now be considered a stranger, with commensurate effects of loss of a previously valued relationship and erosion of trust which is further exacerbated by the processes of the system which disempower both the patient and the professionals working within it. These include the fight to gain access to a HCP and feeling dismissed and devalued, possibly as a result of the bureaucratisation of the primary care system. Alongside the psychosocial impacts, patients experienced significant adverse outcomes for their health that they felt were a result of these changes, which are particularly impactful for those with long-term conditions. In line with the literature, new ways of considering the doctor-patient dyad could be key to improving the experiences of patients with long-term conditions in primary care.

## Data Availability

The raw data supporting the conclusions of this article will be made available by the authors, without undue reservation.
